# Hemodynamic gain index and risk of chronic kidney disease: A prospective cohort study of middle-aged and older men

**DOI:** 10.1007/s11357-024-01184-2

**Published:** 2024-05-06

**Authors:** Setor K. Kunutsor, Jari A. Laukkanen

**Affiliations:** 1 Diabetes Research Centre, University of Leicester, Leicester General Hospital, Gwendolen Road, Leicester, LE5 4WP UK; 2 Institute of Clinical Medicine, Department of Medicine, University of Eastern Finland, Kuopio, Finland; 3 Institute of Public Health and Clinical Nutrition, University of Eastern Finland, Kuopio, Finland; 4 Department of Medicine, Wellbeing Services County of Central Finland, Jyväskylä, Finland

**Keywords:** Hemodynamic gain index, Chronic kidney disease, Exercise testing, Cohort study

## Abstract

The hemodynamic gain index (HGI), a novel non-invasive hemodynamic marker, represents a promising advancement in cardiovascular risk assessment. Cardiovascular disease and chronic kidney disease (CKD) are closely intertwined and share bidirectional relationships. We aimed to assess the association of HGI with CKD risk in a prospective study. Hemodynamic gain index was calculated using heart rate and systolic blood pressure (SBP) responses measured in 1765 men aged 42–61 years with normal kidney function during exercise testing using the formula: [(Heart rate_max_ x SBP_max_)—(Heart rate_rest_ x SBP_rest_)]/(Heart rate_rest_ x SBP_rest_). Multivariable adjusted hazard ratios (HRs) (95% confidence intervals, CIs) were estimated for CKD. Over a median follow-up duration of 25.9 years, 175 CKD cases occurred. In analysis adjusted for established risk factors, a unit (bpm/mmHg) higher HGI was associated with a decreased risk of CKD (HR 0.78, 95% CI 0.65–0.95). Comparing extreme tertiles of HGI, the corresponding adjusted HR (95% CI) for CKD was 0.53 (0.33–0.85). Addition of HGI to a CKD risk prediction model containing established risk factors improved risk discrimination and reclassification (*p*-value for difference in -2 log likelihood = .011; net-reclassification-improvement = 59.37%, *p* = .018; integrated-discrimination-improvement = 0.0064, *p* = .008). Higher HGI is associated with a lower CKD risk and improves the prediction and classification of CKD beyond common established risk factors.

**Supplementary Information:**

The online version contains supplementary material available at 10.1007/s11357-024-01184-2.

## Introduction

Chronic kidney disease (CKD) poses a significant public health burden worldwide, affecting millions of individuals and being associated with substantial morbidity, mortality, and healthcare costs. Major risk factors for CKD encompass hypertension, diabetes, obesity, smoking, and atherosclerotic cardiovascular disease (CVD). Given that CKD is often preventable and manageable when detected early, the search for novel risk indicators becomes paramount. The plentiful health effects of physical activity extend to reducing the risk of CKD as well as delaying its progression [1]. Cardiopulmonary exercise testing (CPX) has emerged as a valuable tool for assessing cardiorespiratory fitness (CRF), a modifiable risk factor that is principally determined through increased aerobic physical activity[2] and shown to be a robust predictor of CVD and overall health [3]. Moreover, emerging evidence indicates a significant relationship between CRF and CKD risk [4]. During CPX, heart rate and blood pressure responses in addition to CRF are core parameters routinely measured. The hemodynamic responses during exercise hold promise as potential markers of cardiovascular health and are gaining attention as valuable prognostic indicators. One such novel hemodynamic marker is the hemodynamic gain index (HGI), which is derived from the combination of exercise heart rate and systolic blood pressure (SBP) responses during CPX [5]. HGI has demonstrated its ability to predict adverse cardiovascular outcomes [6–8], representing a promising advancement in cardiovascular risk assessment. Despite the well-established link between CVD and CKD [9], the association between HGI and CKD risk has yet to be evaluated. Therefore, the primary aim of the current study is to investigate the potential association between HGI and the risk of CKD. We also assessed the extent to which HGI measurements could improve the prediction of CKD using measures of risk discrimination and reclassification.

## Materials and methods

The Kuopio Ischemic Heart Disease (KIHD) population-based prospective cohort study which comprises a representative sample of 2682 men, aged 42–61 yr, drawn from eastern Finland[4] was used for the analysis. Baseline assessments and physical examinations occurred between March 1984 and December 1989. Ethical approval for the study protocol was obtained from the Research Ethics Committee of the University of Eastern Finland, and all participants provided written informed consent. To estimate CRF, peak oxygen uptake was directly assessed using a respiratory gas analyzer (Medical Graphics) during a maximal symptom-limited exercise-tolerance test performed on an electrically braked cycle ergometer [4]. Additionally, electrocardiographic indices, blood pressure (BP), and heart rate were measured both at rest and during the exercise testing phase [7, 8]. The HGI was derived using the following formula: ([Heart rate_max_ x SBP_max_]—[Heart rate_rest_ x SBP_rest_])/(Heart rate_rest_ x SBP_rest_) [5], obtained from the heart rate and SBP responses during the exercise tests. Chronic kidney disease was defined based on the National Kidney Foundation Kidney Disease Outcomes Quality Initiative (KDOQI) guideline, encompassing kidney damage (e.g., albuminuria) or an estimated glomerular filtration rate (eGFR) lower than 60 mL/min per 1.73 m^2^ (or both) for a duration of 3 months or longer [10]. Incident CKD cases that occurred from the commencement of the study until 2014 were included in the analysis. To estimate the hazard ratios (HRs) with 95% CI for CKD, multivariable Cox proportional hazards models were employed. To investigate whether adding information on HGI to established risk factors for CKD is associated with improvement in the prediction of CKD risk, we calculated measures of discrimination (e.g., Harrell’s C-index[11] and difference in -2 log likelihood) and reclassification (net-reclassification-improvement (NRI) and integrated-discrimination-improvement (IDI)) [12, 13]. All statistical analyses were performed using Stata version MP 18 (Stata Corp).

## Results

**Fig. 1:**
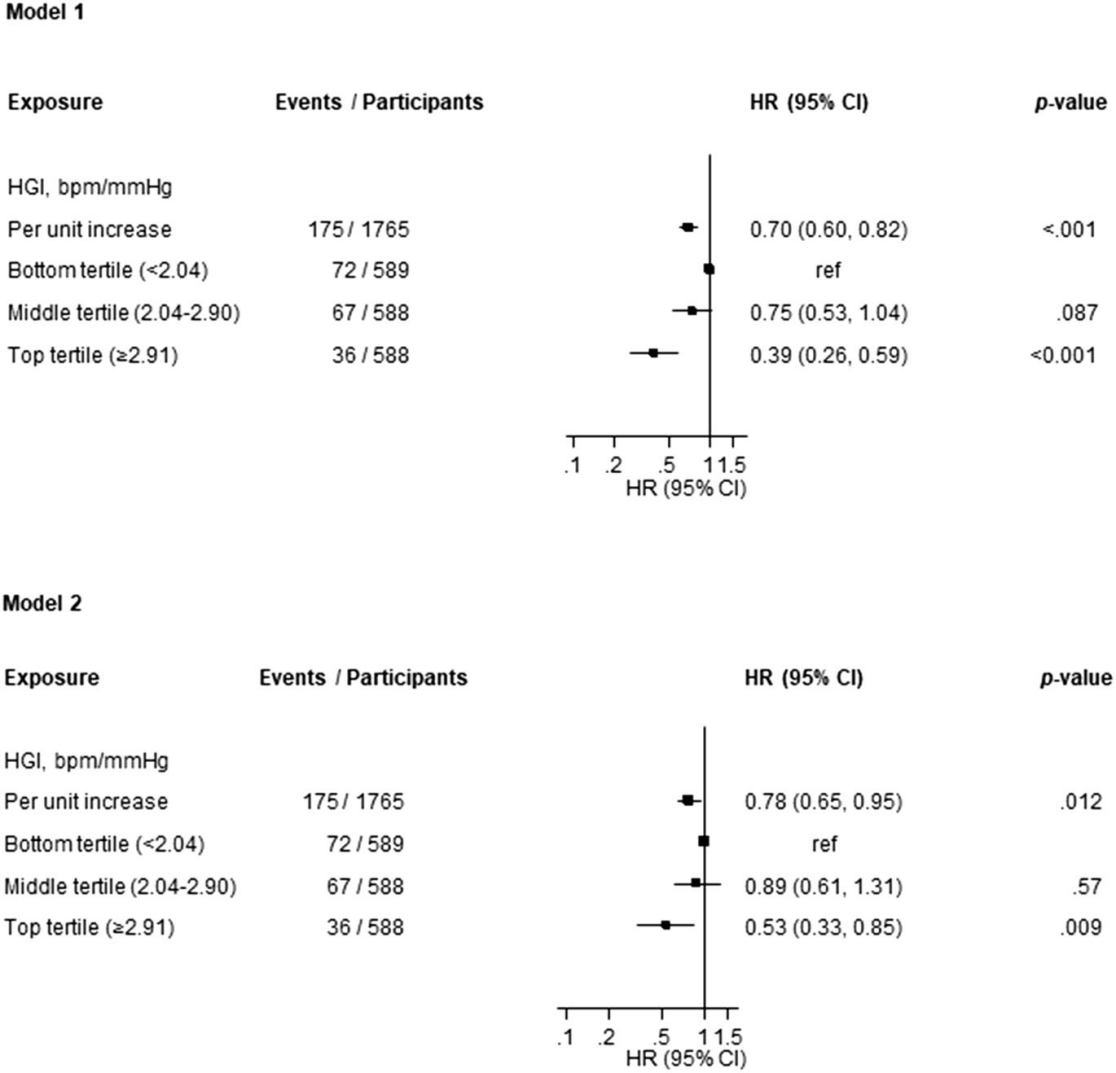
Association of hemodynamic gain index with chronic kidney disease

Among the participants included in the analysis, a total of 1765 men provided complete information on HGI, potential confounding variables, and CKD events (Electronic Supplementary Material 1). At baseline, their mean ± SD age was 53 ± 5 yr, while the mean ± SD HGI was 2.53 ± 1.05 bpm/mmHg (Table 1). Over a median (IQR) follow-up duration of 25.9 (18.0, 28.0) yr, a total of 175 CKD cases were observed. In the age-adjusted analysis, each 1 unit increase in HGI was associated with a significantly lower risk of CKD (HR 0.70, 95% CI 0.60–0.82) (Fig. 1-Model 1), which was attenuated to (HR 0.78, 95% CI 0.65–0.95) on further adjustment for body mass index, total cholesterol, smoking, prevalent type 2 diabetes, hypertension and coronary heart disease, alcohol consumption, socioeconomic status, eGFR and physical activity (Fig. 1-Model 2). Alternatively, comparing individuals in the top tertile of HGI to those in the bottom tertile, the corresponding adjusted HRs (95% CIs) for CKD were 0.39 (0.26–0.59) and 0.53 (0.33–0.85), respectively. A CKD risk prediction model containing traditional risk factors yielded a C-index of 0.6500 (95% CI: 0.6022 to 0.6978). After addition of information on HGI, the C-index was 0.6615 (95% CI: 0.6143 to 0.7088), representing a modest increase of 0.0115 (95% CI: -0.0042 to 0.0273; *p* = 0.15). The -2 log likelihood was significantly improved on addition of HGI to the risk model (*p* for comparison = 0.011). The continuous NRI and IDI were 59.37% (95% CI: 10.29 to 108.45; *p* = 0.018) and 0.0064 (0.0016 to 0.0111; *p* = 0.008), respectively.

**Table 1:** Baseline characteristics of study participants overall and by chronic kidney disease

Characteristics	Overall (n = 1765) Mean ± SD or median (IQR)	With CKD (n = 175) Mean ± SD or median (IQR)	Without CKD (n = 1590) Mean ± SD or median (IQR)
Hemodynamic gain index, bpm/mmHg	2.53 ± 1.05	2.29 ± 0.94	2.56 ± 1.06
Resting heart rate on bicycle, bpm	62 ± 11	62 ± 11	62 ± 11
Peak heart rate on bicycle, bpm	155 ± 25	148 ± 28	156 ± 24
Resting SBP on bicycle, mmHg	150 ± 22	154 ± 24	150 ± 22
Peak SBP on bicycle, mmHg	204 ± 27	205 ± 30	204 ± 27
Age, yr	53 ± 5	54 ± 5	53 ± 5
Socioeconomic status	8.4 ± 4.3	9.4 ± 4.3	8.3 ± 4.2
Body mass index, kg/m^2^	26.9 ± 3.4	27.8 ± 3.8	26.8 ± 3.4
Alcohol consumption, g/week	32.9 (6.4, 95.6)	32.0 (6.3, 96.0)	33.0 (6.4, 95.6)
Physical activity, KJ/day	1163 (620, 1914)	1215 (565, 1826)	1162 (622, 1938)
Current smoking	568 (32.2%)	49 (28.0)	519 (32.6%)
History of type 2 diabetes	63 (3.6%)	8 (4.6%)	55 (3.5%)
History of hypertension	526 (29.8%)	60 (34.3%)	466 (29.3%)
History of coronary heart disease	415 (23.5%)	54 (30.9%)	361 (22.7%)
Total cholesterol, mmol/l	5.93 ± 1.09	6.04 ± 1.15	5.92 ± 1.09
Estimated GFR, ml/min/1.73 m^2^	88.2 ± 17.0	87.1 ± 15.7	88.3 ± 17.2

BMI, body mass index; CKD, chronic kidney disease; GFR, glomerular filtration rate; IQR, interquartile range; SD, standard deviation; SBP, systolic blood pressure

Socioeconomic status was generated as a summary index that combined factors such as income, education, occupational prestige, material standard of living and housing conditions. The composite index ranged from 0 to 25, with higher values indicating lower socioeconomic status

## Discussion

Higher HGI demonstrated a strong association with a lower risk of CKD, even after accounting for several well-established risk factors. Furthermore, addition of information on HGI to a model containing traditional risk factors for CKD was associated with significant improvements in the discrimination and reclassification of long-term CKD. The significance of the association of HGI with CKD risk lies in the fact that hemodynamic responses to exercise, such as heart rate and blood pressure, have been recognized as independent predictors of vascular disease and essential determinants of prognosis [14]. The index reflects the compliance of the vasculature and the cardiovascular system's ability to generate sufficient blood flow to meet physiological demands during maximal aerobic exercise [5]. Higher HGI levels during exercise have been associated with better cardiac function and predict adverse cardiovascular outcomes [6–8]. Since CVD and CKD are closely intertwined and share complex and bidirectional relationships, a more robust cardiovascular system, represented by higher HGI, may confer protection against CKD development. The implications of these findings are significant in the context of CKD prevention and management. The identification of HGI as a strong and independent risk indicator and predictor of CKD provides a promising easily accessible and valuable tool for long-term risk stratification. Early identification of individuals at increased CKD risk using HGI could facilitate targeted interventions, potentially reducing the burden of CKD and its associated complications. Further research and validation studies are warranted to establish HGI's clinical value in risk stratification and its potential implications for CKD prevention.

The strengths of this study include its robust cohort design, a relatively large sample size, long-term follow-up with zero loss to follow-up, comprehensive adjustment for multiple confounding factors and assessment of risk prediction using established methods and measures. However, certain limitations should be acknowledged. Firstly, the study population consisted of middle-aged and older Finnish men, limiting the generalizability of the findings to women and other populations. Furthermore, the observational design poses potential concerns regarding reverse causation and regression dilution, necessitating cautious interpretation of the results.

## Conclusion

The current study contributes valuable insights into the relationship between HGI and CKD risk. HGI is a strong risk indicator and predictor of CKD and represents a simple and non-invasive marker for assessing CKD risk.

### Supplementary Information

Below is the link to the electronic supplementary material.

Supplementary file1 (DOCX 57 KB)

## Data Availability

Restrictions apply to the availability of the outcome data due to recent Finnish legislation concerning the use of national social and health registers in research.

## Declarations

The KIHD study was conducted in accordance with the ethical standards set forth in the Helsinki Declaration (1983). Participants in the study were included after obtaining their written and informed consent for the study. The institutional review board of the University of Kuopio and Kuopio University Hospital, Kuopio, Finland (License number 143/97) approved the study protocol.
