# Supplementary material for: Hemodynamic gain index and risk of chronic kidney disease: A prospective cohort study of middle-aged and older men
Source: GeroScience. 2024 May 6;46(5):5211–5. doi: 10.1007/s11357-024-01184-2 (PMC11335700; doi:10.1007/s11357-024-01184-2)
Supplement: Supplementary file 1 — Supplementary file1 (DOCX 57 KB) [file 11357_2024_1184_MOESM1_ESM.docx]

**Electronic Supplementary Material 1.** Flow of study participants

HGI, hemodynamic gain index; CKD, chronic kidney disease
